# *Candida albicans*/Macrophage Biointerface on Human and Porcine Decellularized Adipose Matrices

**DOI:** 10.3390/jof7050392

**Published:** 2021-05-17

**Authors:** Mónica Cicuéndez, Laura Casarrubios, María José Feito, Iratxe Madarieta, Nerea Garcia-Urkia, Olatz Murua, Beatriz Olalde, Nerea Briz, Rosalía Diez-Orejas, María Teresa Portolés

**Affiliations:** 1 Departamento de Bioquímica y Biología Molecular, Facultad de Ciencias Químicas, Instituto de Investigación Sanitaria del Hospital Clínico San Carlos (IdISSC), Universidad Complutense de Madrid, 28040 Madrid, Spain; mcicuendez@ucm.es (M.C.); laura.casarrubios.molina@gmail.com (L.C.); mjfeito@ucm.es (M.J.F.); 2 TECNALIA, Basque Research and Technology Alliance (BRTA), E20009 Donostia-San Sebastian, Spain; iratxe.madarieta@tecnalia.com (I.M.); nerea.garcia@tecnalia.com (N.G.-U.); olatz.murua@tecnalia.com (O.M.); nerea.briz@tecnalia.com (N.B.); 3 Departamento de Microbiología y Parasitología, Facultad de Farmacia, Universidad Complutense de Madrid, 28040 Madrid, Spain; 4 CIBER de Bioingeniería, Biomateriales y Nanomedicina, CIBER-BBN, 28040 Madrid, Spain

**Keywords:** *Candida albicans*, macrophage, extracellular matrix, decellularization, immunocompetence, phagocytosis

## Abstract

Macrophages, cells effective in sensing, internalizing and killing *Candida albicans*, are intertwined with the extracellular matrix (ECM) through different signals, which include the release of specific cytokines. Due to the importance of these interactions, the employment of in vitro models mimicking a fungal infection scenario is essential to evaluate the ECM effects on the macrophage response. In this work, we have analyzed the effects of human and porcine decellularized adipose matrices (DAMs), obtained by either enzymatic or organic solvent treatment, on the macrophage/*Candida albicans* interface. The present study has allowed us to detect differences on the activation of macrophages cultured on either human- or porcine-derived DAMs, evidencing changes in the macrophage actin cytoskeleton, such as distinct F-actin-rich membrane structures to surround the pathogen. The macrophage morphological changes observed on these four DAMs are key to understand the defense capability of these cells against this fungal pathogen. This work has contributed to the knowledge of the influence that the extracellular matrix and its components can exert on macrophage metabolism, immunocompetence and capacity to respond to the microenvironment in a possible infection scenario.

## 1. Introduction

*Candida albicans* is a commensal fungus found frequently as part of the normal mucosa in different host niches [1]. This opportunistic fungal pathogen can initiate a variety of recurring superficial and invasive infections when the host immunocompetence decrease [2,3]. *C. albicans* is a dimorphic fungus having either an ovoid-shaped budding yeast form or pseudohyphae with chains of elongated buds and true hyphae consisting of chains of thin cells with parallel cell walls [4,5]. Adhesion of *C. albicans* to host cells is essential for colonization and survival in the host [6]. Hyphae are generally considered to be the more adherent morphology of *C. albicans*, being induced after the first contact with a host cell surface [7]. The immune cells, acting as the first line of defense against this fungal pathogen, are professional phagocytes, including polymorphonuclear neutrophils (PMNs), monocytes/macrophages and dendritic cells. Macrophages exhibit great functional plasticity with a spectrum of different phenotypes between two extremes: the M1 or pro-inflammatory phenotype and the M2 or reparative phenotype, characterized by specific markers and cytokines [8]. TNFα is a pro-inflammatory cytokine that regulates many aspects of macrophage function and has been shown to be one of the most abundant early mediators in inflamed tissue. Among its various functions is its pivotal role in organizing the production of the pro-inflammatory cytokine cascade [9]. IL-6 also plays a role in the body’s defense against infection and in many regenerative processes [10,11]. Macrophages are effective cells in sensing, internalizing and killing *C. albicans*. The macrophage/fungus interaction requires the cytoskeletal reorganization in these phagocytic cells, producing distinct F-actin-rich membrane structures to surround the pathogen [12,13]. The response of *C. albicans* to macrophages has been extensively studied and different escape strategies after phagocytosis have been described [14,15]. In this sense, filamentation of phagocytosed *C. albicans* cells causes piercing of macrophage membranes, macrophage killing and escape. Moreover, hyphal cells are difficult to be engulfed by macrophages [16,17]. Thus, morphogenesis has a great impact on the macrophages–fungus interaction, and it has been defined as an important mechanism of virulence.

It has recently been shown that macrophage metabolism plays a key regulatory role in macrophage activation and biological function, influencing signal transduction and gene regulation and leading to the appropriate response to a particular stimulus or environment [18]. Thus, it has been evidenced that glucose metabolism is redirected to allow *de novo* lipogenesis to occur during the inflammatory response and that the fatty acid esterification and storage in lipid droplets (LDs) prevent proinflammatory macrophage activation [19,20]. In the reparative response, interleukin-4 (IL-4) promotes fatty acid oxidation, producing acetyl-CoA that can be used to promote histone acetylation and expression of IL-4 inducible genes. Thus, fatty acid oxidation is linked to the M2 state, which is driven by IL-4 and IL-13 produced during infections to regulate tissue repair. Cholesterol also has a key role in the macrophage response against pathogens [21].

The extracellular matrix (ECM), through its interaction with macrophages, controls specific responses that are key for tissue homeostasis and aspects related to cell phenotype [22]. Numerous biomaterials for tissue engineering and regenerative medicine are based on the decellularized extracellular matrix (DAM) to promote rapid healing at the implant site, reduce adverse inflammatory responses and prevent implant rejection [23,24]. This kind of biomaterial provides mechanical support and biochemical signals to enhance cell attachment and modulate cell behavior, regulating cell phenotype and function in development, homeostasis and response to infection. In vitro assessment of the macrophage behavior in contact with decellularized material is essential to understand how the organism will respond to these biomaterials after implantation in vivo. The polarization of macrophages towards M1 or M2 phenotypes after interaction with the implant will determine its integration into the tissue [25].

Human and porcine adipose tissue (AT) has high potential as an abundant source of ECM to prepare scaffolds designed for the growth, differentiation and phenotypic preservation of different cell types [26,27]. The techniques commonly used to decellularize tissues include physical, chemical and biological agents [28,29]. The employment of coating cell culture plates with purified ECM components provides a very useful in vitro model to mimic the dynamics and composition of the ECM [30]. On the other hand, the study of cell adhesion has been widely explored for many important biomedical and tissue engineering objectives [31,32]. Adhesion is an essential process in cell communication and regulation and is involved in tissue maintenance and development [33]. Cell adhesion is defined as the ability to directly interact with different surfaces, such as the ECM, or with other cells. In this context, it is important to understand the influence that the ECM and its components can exert on macrophage metabolism, immunocompetence and capacity to respond to the microenvironment in a possible infection scenario.

In this work, we have prepared coatings of four DAMs obtained by two different treatments from human and porcine AT, following a methodology previously published by our group [34], and analyzed their effect on the macrophage/*Candida albicans* interface. Thus, the objective of this study is to know how macrophages exposed to biomaterials prepared with these DAMs would respond in a fungal infection scenario. Different cell biological and biochemical parameters of the macrophages and fungus have been analyzed by confocal microscopy after exposure to these DAMs.

## 2. Materials and Methods

### 2.1. Human and Porcine Adipose Tissue Decellularization

Porcine AT was harvested from a local food company (JAUCHA S.L., Navarra, Spain) and human AT from Biopredict International (Saint Grégoire, France), according to the French Ministry of Higher Education and Research permission AC-2013-1754. Both porcine and human ATs were cleaned, creamed using a beater and stored at −20 °C.

Tissues were decellularized following the methodology previously published by our group [34]. Briefly, protein pellets were obtained by homogenization (Polytron PT3100) at 12,000 rpm for 5 min, centrifugation (5000 rpm for 5 min) with ultrapure water and manual discarding of lipids conserving the protein pellets. Afterwards, the protein pellets were treated by two different methods. In the first protocol, tissues were treated with two enzymes, lipoprotein lipase (Merck Life Science SL, Madrid, Spain) and Benzonase^®^ (Emprove^®^-bio, Merck Life Science SL, Madrid, Spain). In the second method, tissues were treated with isopropanol (Merck Life Science SL, Madrid, Spain) and 1% (*v*/*v*) triton x-100 and 0.1% (*v*/*v*) ammonium hydroxide (Merck Life Science SL). Each step was followed by cleaning with Phosphate Buffer Saline (PBS, Merck Life Science SL) supplemented with 1% (*v*/*v*) antibiotic antimycotic solution (Gibco-BRL, Paisley, UK) and a last wash with Ultrapure milli Q water. The decellularized adipose matrices (DAMs) obtained by the enzymatic method were identified as porcine and human DAM1 (pDAM1 and hDAM1, respectively) and those obtained by the organic solvent method were identified as porcine and human DAM2 (pDAM2 and hDAM2, respectively). In order to create a fine-grained powder suitable for processing, all the DAMs were milled using a mixer mill (Retsch MM400, Haan, Germany) and conserved at 4 °C in a vacuum desiccator.

### 2.2. Characterization of DAMs and Coating Preparation

DAMs were characterized for remnant DNA and lipids as well as for protein composition following the methodology previously described by our group [34]. Briefly, DNA was extracted from the DAMs (QiAmp kit, Qiagen, Madrid, Spain) and absolute quantification was done by quantitative real-time polymerase chain reaction (qRT-PCR) and based on standard curves of known human and porcine DNA samples. The total lipid content was determined in the DAMs by liquid chromatography coupled with Q-TOF mass spectrometry (UPLC/Q-TOF MS). Briefly, the extracts were injected into a Waters column (Acquity UPLC HSS T3 1.8 µm, 100 mm × 2.1 mm) and heated to 65 °C. The mobile phases consisted of acetonitrile and water with 10 mM ammonium acetate (40:60, *v*/*v*) (phase A) and acetonitrile and isopropanol with 10 mM ammonium acetate (10:90, *v*/*v*) (phase B). The flow rate was 0.5 mL/min and the injection volume was 5 µL. UHPLC-MSE data were acquired on a SYNAPT G2 HDMS, with a quadrupole time of flight (Q-ToF) configuration and (Waters) equipped with an electrospray ionization (ESI) source. The protein composition was analyzed by liquid chromatography with tandem mass spectrometry (LC-MS/MS) in native ATs and DAMs. Briefly, samples were homogenized in 8 M urea using a Precellys^®^24 homogenizer (Bertin Technologies, Brussels, Belgium), sonicated for 3 min and clarified by centrifugation at 16,000× *g* for 10 min. Proteins were reduced (5 mM DTT), alkylated (15 mM iodoacetamide) and digested with trypsin (0.01 µg/µL). The resulting peptides were desalted using C-18 Micro SpinColumns (Harvard Apparatus, Holliston, MA, USA). LC-MS/MS analysis was performed using a Q Exactive (Thermo Fisher Scientific, Madrid, Spain).

Milled DAMs were used to prepare coatings following the methodology previously published by our group [34]. Briefly, milled DAMs were dissolved in 1M acetic acid (Panreac Quimica SLU, Barcelona, Spain); the solutions were added to polystyrene plates and glass coverslips (5 min room temperature), washed (3 × 5 min) with ultrapure Milli-Q water (Millipore) and dried (room temperature, 2 h). Effectively coated total protein was characterized by Pierce Micro BCA Protein Assay Kit (Thermo Fisher Scientific, Madrid, Spain).

### 2.3. Culture of RAW-264.7 Macrophages on DAMs

RAW-264.7 macrophages were cultured at a cell density of 10^5^ cells/mL for 24 h at 37 °C under a 5% CO_2_ atmosphere in culture plates of 6-well culture plates coated with hDAM1, hDAM2, pDAM1 and pDAM2. The culture medium was Dulbecco’s Modified Eagle Medium (DMEM, Gibco-BRL, Paisley, UK) supplemented with 10% fetal bovine serum (FBS, Gibco-BRL, Paisley, UK), 1 mM L-glutamine (BioWhittaker Europe, Verviers, Belgium), penicillin (200 μg/mL, BioWhittaker Europe, Verviers, Belgium) and streptomycin (200 μg/mL, BioWhittaker Europe, Verviers, Belgium).

### 2.4. Morphological Studies of Macrophages by Scanning Electron Microscopy (SEM)

For scanning electron microscopy studies, RAW-264.7 macrophages were cultured on circular glass coverslips coated with each of the four DAMs under the abovementioned culture conditions. Then, cells were fixed with glutaraldehyde (2.5% in PBS) for 45 min. Sample dehydration was performed by slow water replacement using a series of ethanol solutions (30, 50, 70 and 90%) for 15 min with a final dehydration in absolute ethanol for 30 min, allowing samples to dry at room temperature and under vacuum. Afterwards, the samples were mounted on stubs, coated in vacuum with gold-palladium and observed with a JEOL JSM-6400 scanning electron microscope.

### 2.5. Candida albicans Strain

The *C. albicans* strain used in this study was the CAF2-dTOM2 derived from SC5314 expressing a red fluorescent protein (RFP-*Candida albicans*) [35], kindly provided by Dr. D. Prieto and Dr. J. Pla. This red fluorescent strain was synthesized by codon optimization of the DsRed-derived RFP dTomato gene [36] using the tetracycline-dependent integrative plasmid pNIM1R. Expression in *C. albicans* was also detectable in SD plates as pink-reddish-colored colonies after two days of growth [37]. The yeast strain was short-term stored at 4 °C and grown at 37 °C in YPD medium (2% glucose, 2% peptone, 1% yeast extract) plus amino acids and chloramphenicol (10 µg/mL) for 48 h.

### 2.6. Confocal Microscopy Studies of Candida albicans/Macrophage Biointerface on Human and Porcine DAMs

RAW-264.7 macrophages were cultured on circular glass coverslips coated with each of the four DAMs under the abovementioned culture conditions and exposed to RFP-*Candida albicans* at a macrophage–fungus ratio of 1:5 (multiplicity of infection 5, MOI 5). Controls with macrophages without *C. albicans* infection were carried out in parallel. After 45 min of interaction at 37 °C under a 5% CO_2_ atmosphere, non-ingested and unbound *Candida* cells were removed by washing three times with ice-cold PBS and the RFP-*Candida albicans*/RAW-264.7 macrophage interface was observed by confocal microscopy. With this objective, cells were fixed with paraformaldehyde (3.7%) and permeated with 500 μL of Triton-X100 (0.1% in PBS). After 20 min of incubation with BSA (1% in PBS), samples were incubated for 20 min with FITC-phalloidin 1:40 (100 μL) to stain the F-actin filaments, washed with PBS and stained with 100 μL of 3 μM DAPI. Finally, samples were observed by using an Olympus FV1200 confocal laser scanning microscope. The fluorescence of RFP-expressing *C. albicans* was excited at 488 nm and measured at 584/663 nm. FITC fluorescence was excited at 488 nm and measured at 491–586 nm. DAPI fluorescence was excited at 405 nm and measured at 409/468 nm. To quantify the different cell parameters (filopodia length and chromatin condensation of RAW 264.7 macrophages), at least 10 fields were observed in the different samples and 500 macrophages were counted in each condition.

### 2.7. Detection of TNF-α and IL-6 as Inflammatory Cytokines

The amounts of TNF-α and IL-6 secreted by RAW-264.7 macrophages were quantified in the culture medium by enzyme-linked immunosorbent assay (ELISA, Gen-Probe, Diaclone, Besançon, France) according to the manufacturer’s instructions.

### 2.8. Statistics

Data are expressed as the mean ± standard deviation of a representative of three experiments carried out in triplicate. Statistical analysis was performed using the Statistical Package for the Social Sciences (SPSS) v. 22 software. Statistical comparisons were made by analysis of variance (ANOVA). A Scheffé test was used for post-hoc evaluations of the differences among groups. In all the statistical evaluations, *p* ˂ 0.05 was considered as statistically significant.

## 3. Results and Discussion

### 3.1. Characterization of DAMs

The goal of the decellularization is the effective removal of cellular and nuclear components while preserving the composition and structure properties of the native ECM proteins [38]. Specifically, for AT, an effective delipidation is also mandatory. Our group has previously published the effective decellularization and delipidation of porcine and human AT by two different methods, enzymatic (method 1) and organic solvent (method 2). [34]. All the resultant DAMs (hDAM1, pDAM1, hDAM2 and pDAM2) meet the decellularization criteria stablished by the research community (less than 50 ng DNA/mg dry weight) and the delipidation was also effective. Porcine-derived DAMs showed much higher values of remnant lipids than human-derived DAMs. Interestingly, DAMs obtained from AT decellularized by the enzymatic treatment with lipoprotein lipase and Benzonase^®^ exhibited a lower content of triacylglycerols than DAMs processed by treatment with the organic solvent (isopropanol). These results evidenced the specific action of lipoprotein lipase on triacylglycerols contained in adipocytes, leading to low levels of this kind of lipid in hDAM1 and pDAM1.

**Figure 1:**
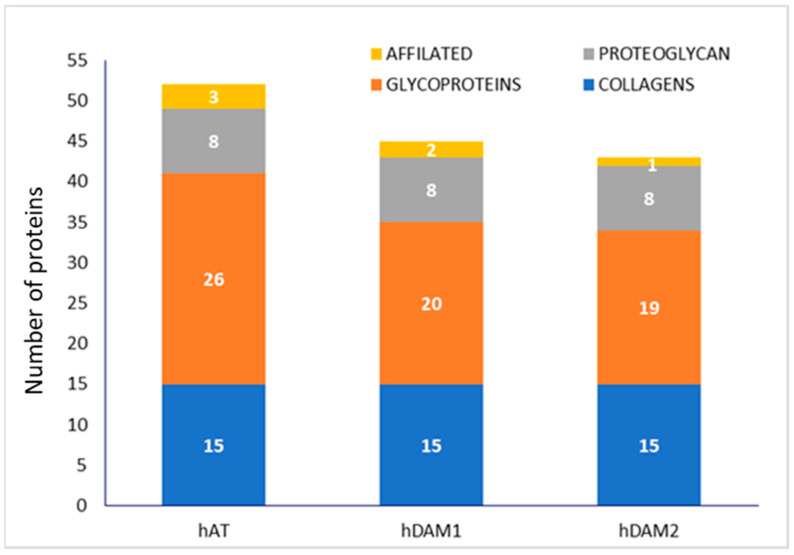
Number of adipose ECM proteins classified by groups in human native AT (hAT) and human DAMs obtained by Method 1 (hDAM1) and Method 2 (hDAM2).

**Figure 2:**
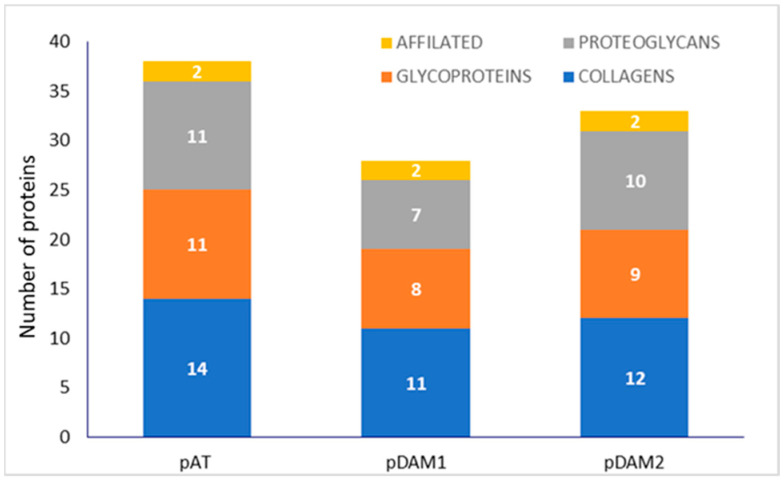
Number of adipose ECM proteins classified by groups in porcine native AT (pAT) and porcine DAMs obtained by Method 1 (pDAM1) and Method 2 (pDAM2).

Related to the protein composition of DAMs, for an easier comprehension, a previously published complete proteomic analysis [34] has been resumed, with clustering by protein groups (collagens, proteoglycans, glycoproteins and affiliated proteins), and is shown schematically for native and DAMs for human (Figure 1) and porcine (Figure 2) tissue.

Human-derived DAMs (hDAM1 and hDAM2) conserved all the collagens and proteoglycans presented in the native tissue (hAT). However, some glycoproteins and affiliated proteins were not conserved after the decellularization. Besides, the enzymatic method conserved more ECM proteins than Method 2. Related to porcine-derived DAMs (pDAM1 and pDAM2), some collagens, glycoproteins and proteoglycans have not been conserved after the decellularization and more ECM proteins were conserved by the organic solvent method.

All the DAMs were successfully used to coat the surface of the 6-well polystyrene plate and glass coverslips, as was confirmed in previously published work of this group by the quantification of the effectively coated total protein [34].

### 3.2. Adhesion of Macrophages on DAMs

Macrophages, as essential cells of the innate immune system, adopt a variety of functional phenotypes, depending on the signals of their environment. The response of these phagocytes can be tuned by the interaction with the surface of biomaterials based on decellularized ECM [39,40]. Studies from the biomaterial community have contributed significantly to the knowledge about how the physical and chemical characteristics of biomaterial influence macrophage functioning, such as the secretion of inflammatory cytokines [41]. Cell adhesion will be the first event in the interaction between the biomaterial surface and macrophages, playing a major role in sensing mechanical cues and transducing them into biological signals that will regulate cell function. This recognition involves the participation of different integrins, which bind to the ECM and connect the extracellular environment with the intracellular cytoskeleton, playing a central role in morphological changes, motility, proliferation and differentiation [32].

**Figure 3:**
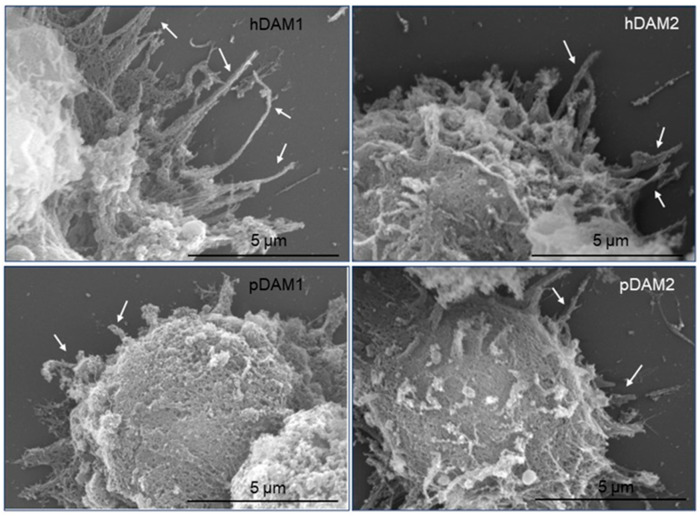
Representative SEM images of the cellular morphology of RAW-264.7 macrophages after 24 h of culture on human (h) and porcine (p) DAMs obtained by the enzymatic method (hDAM1and pDAM1) and organic solvents treatment (hDAM2 and pDAM2). Characteristic filopodia are indicated by arrows.

In order to evaluate the effects of the biochemical composition of the four DAMs obtained in this work (hDAM1, hDAM2, pDAM1 and pDAM2) on the macrophage adhesion, we carried out scanning electron microscopy (SEM) studies with macrophages cultured 24 h on coatings prepared from these DAMs. Figure 3 displays the cellular morphology of the macrophages that adhered to the surface of these DAMs, obtained by the enzymatic method (hDAM1and pDAM1) and organic solvents treatment (hDAM2 and pDAM2). The images show the macrophage morphology after adhesion to these coatings and also evidence some differences concerning the length of the filopodia emitted on the different substrates. Thus, while macrophages cultured on human DAMs (hDAM1 and hDAM2) exhibit long filopodia, those grown on porcine DAMs (pDAM1 and pDAM2) are characterized by shorter filopodia, indicated by arrows in the following images.

**Figure 4:**
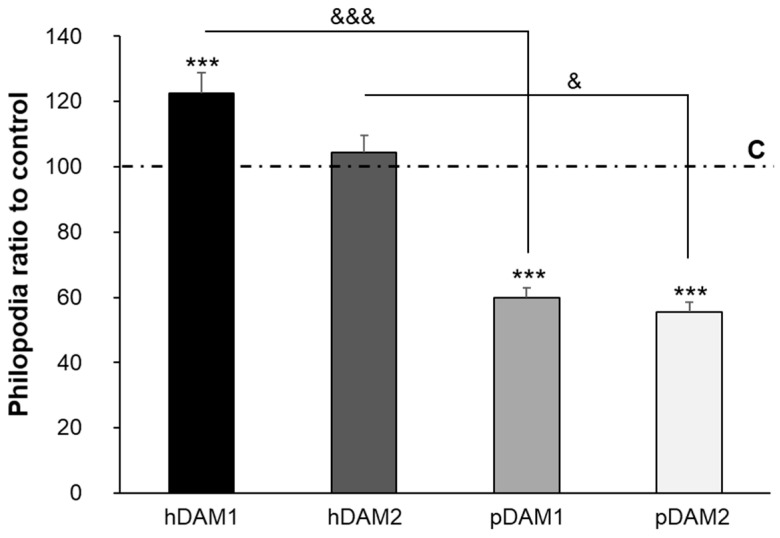
Filopodia length of RAW-264.7 macrophages on human (h) and porcine (p) DAMs obtained by enzymatic method (hDAM1, pDAM1) and organic solvent treatment (hDAM2, pDAM2). The values, obtained after at least 150 *C. albicans* cells scored per experiment, are compared to control macrophages cultured on glass coverslips (horizontal line C). Statistical significance: ***: *p* < 0.005 (in comparison to the control); &: *p* < 0.05; &&&: *p* < 0.005 (hDAM1 vs. pDAM1, hDAM2 vs. pDAM2).

Filopodia are thin and actin-rich protrusions of the plasma membrane that function to explore the cell environment, thus serving as pioneers during outgrowth and cell migration. The initiation and elongation of filopodia depend on the precisely regulated polymerization and crosslinking of actin filaments. Moreover, extensive studies have identified key actin-associated proteins that have an important role during the initiation and elongation of filopodia in different cell types. These molecules include signaling proteins, actin-crosslinking proteins and factors that regulate actin filament nucleation and elongation, among others [42]. Particularly, when macrophages find either a pathogen or an inert particle, the filopodia will bind to them and then retract towards the macrophage cell body, transforming themselves into an actin-based membrane structure called the phagocytic cup [43,44,45]. Thus, considering the key role of filopodia in the macrophage recognition of their microenvironment, we carried out a quantitative study of the filopodia length of macrophages cultured on these human and porcine DAMs compared to control macrophages cultured on glass coverslips. Figure 4 evidences that the filopodia length of macrophages cultured on hDAM1 and hDAM2 was longer than in control cells (horizontal line C on the graph). However, this difference was statistically significant only in the case of hDAM1. Conversely, macrophages cultured on both pDAM1 and pDAM2 samples presented a statistically significant shorter filopodia compared to the control. It is also important to note that when comparing this parameter in human and porcine matrices decellularized by the same method, i.e., hDAM1 vs. pDAM1 and hDAM2 vs. pDAM2, there were significant and very pronounced differences. These results evidence that the type of species from which the adipose tissue derives is the main factor affecting filopodia length.

As has previously been mentioned, the length of the filopodia can be influenced by the cell microenvironment and it is directly related to cell adhesion [32]. In this context, Table 1 includes a selection of ECM proteins presented in the DAMs that might be involved in the macrophage adhesion process and were differentially preserved after both decellularization methods.

Human-derived DAMs, hDAM1 and hDAM2, have a higher number of different types of collagen, laminins, fibulins and EMILIN proteins compared to porcine-derived DAMs, pDAM1 and pDAM2 (Figure 1 and Figure 2). A large amount of scientific data indicate that all these proteins play a key role in different macrophage cellular functions. Collagens are the most abundant ECM proteins that confer tensile strength to tissues, and they are characterized by the repetition of the sequence Gly-X-Y in the polypeptide chain, where X and Y can be any amino acid, but are frequently proline and hydroxyproline. Interestingly, it was demonstrated that macrophages do not adhere well to native type I collagen [46,47]. However, this collagen undergoes cleavage by the fibroblast activation protein-α (FAP); this fact promotes macrophage attachment and spreading, and these interactions are specifically mediated by macrophage class A scavenger receptors (SR-A/CD204) [48]. These results demonstrate once again the influence of the state and composition of the ECM on cell adhesion. On the other hand, collagen IV is an indispensable constituent for the structural integrity of basement membranes [49] and it was shown that macrophages directly might remodel these native barriers [50]. Moreover, results published by Etich et al. indicate that macrophages are also a source of collagen IV and might contribute to the integrity of vascular and skin basement membranes [51].

Laminins are trimeric proteins that constitute the other main structural element of basement membranes and are found in the glycoprotein category within the macrophage matrisome. Apart from their structural role, they also impact the migration and functions of immune cells [52]. Laminins can act as ligands that bind cell membrane receptors (mainly integrins), initiating integrin-mediated signaling. Thus, macrophage adhesion to laminin seems limited to activated macrophages and is mediated by integrin α6β1 [53]. However, the precise details of the effects of laminins on immune cells and the molecular pathways involved are largely unknown. Most of the experiments regarding the laminins–immune cells interaction involved laminins coated on plates as the more suitable in vitro model [52]. The laminin α5 chain is also expressed in both the vascular and the interfollicular epidermis basement membrane and promotes angiogenesis and re-epithelialization [54]. On the other hand, the laminin α3 chain is known to bind growth factors from the VEGF/PDGF, fibroblast growth factor (FGF), bone morphogenetic protein (BMP) and neurotrophin families, promoting tissue regeneration and remodeling upon release [55].

EMILINs are glycoproteins of elastic fibers and are found in regions where elastin and fibrillin microfibrils are in close contact. On the other hand, it is thought that macrophages may produce EMILINs to regulate elastic fiber formation [56].

Regarding fibulin-5 (FBLN5), this protein is essential for elastic fiber formation and for stabilization and organization of elastic fibers in skin cells. In addition, fibulin-5 promotes adhesion of endothelial cells through interaction with integrins and inhibits angiogenesis and endothelial cell activities by antagonizing VEGF signaling independent of its integrin-binding properties [57]. Finally, it has recently been shown that von Willebrand factor also binds to macrophage-specific scavenger-receptor SR-AI [58].

It is important to note that proteolytic fragments of ECM components provide signals affecting cell adhesion, shape, migration, proliferation/survival and differentiation of diverse immune cells [59,60]. Therefore, the macrophage filopodia length and, consequently, cell adhesion on the four decellularized adipose matrices, could be related to the presence or absence of these proteins (Table 1). In summary, the finding in hDAMs of the different types of collagen (Collagen α-1, α-2 and α-3), laminins (laminin α-β-γ), fibulins and von Willebrand factor suggest their relationship with the longer filopodia observed in macrophages cultured on these surfaces (Figure 3 and Figure 4).

### 3.3. Candida albicans/Macrophage Biointerface on Human or Porcine DAMs

Macrophages are essential cells for the homeostasis and immune defense of the organism, due to their ability to engulf and ingest external pathogens, dead cells or many other types of particles. These phagocytic cells play an important role against invading microorganisms due to their high microbicide capacity. Phagocytosis is a process where the engulfment of pathogens requires actin cytoskeletal remodeling to produce distinct F-actin-rich membrane structures. Thus, macrophage filopodia act as microenvironment sensors and, after finding a pathogen, will surround it, transforming this high-actin structure into a phagocytic cup [12,13]. Considering the previously observed differences in macrophage filopodia length after culture on the four DAMs, we have analyzed the involvement of these protrusions during *Candida albicans*/macrophage interaction.

**Figure 5:**
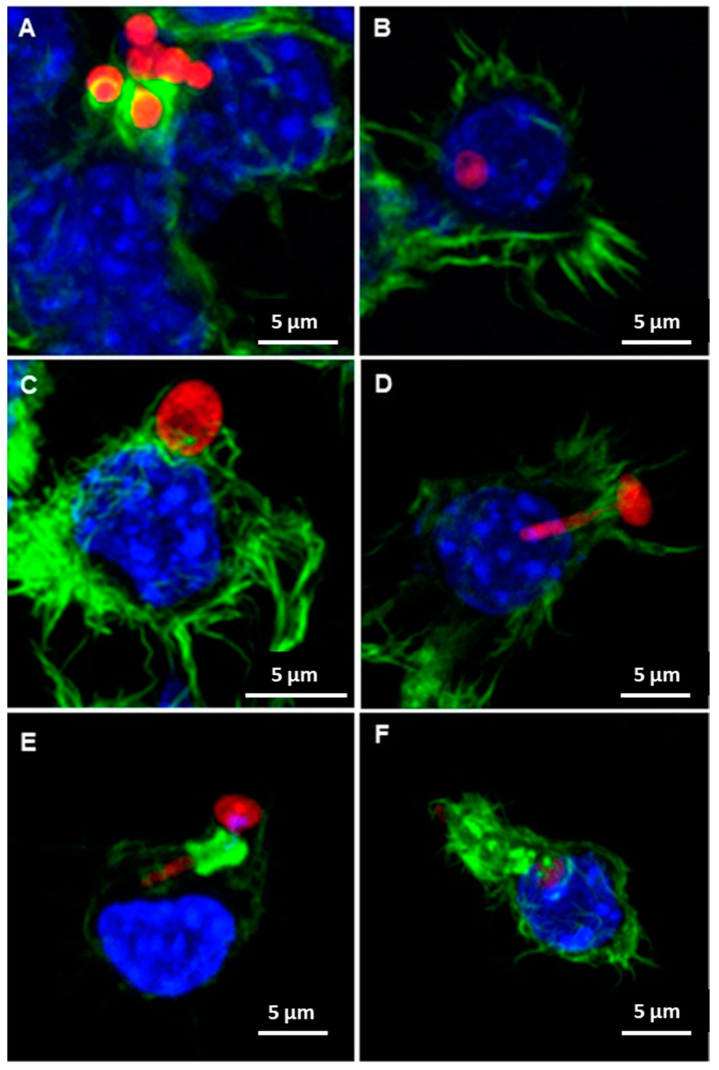
Representative confocal microscopy images of the *Candida albicans*/macrophage biointerface on the control surface (**A**,**B**) and human (h) and porcine (p) DAMs obtained by the enzymatic method and organic solvent treatment (**C** = hDAM1, **D** = hDAM2, **E** = pDAM1 and **F** = pDAM2). Nuclei were labeled in blue with DAPI, and the actin cytoskeleton was labeled in green with FITC-phalloidin. RFP-*C. albicans* (in red).

Figure 5 shows confocal microscopy descriptive images of the phagocytic cells cultured on control surfaces, human and porcine DAMs (hDAM1, hDAM2, pDAM1 and pDAM2), and then infected with the fungal pathogen *Candida albicans*.

Figure 5A,B correspond to macrophages cultured on the control surface (glass coverslips). These representative images show the phagocytic cups formed by macrophages to engulf *C. albicans*, in agreement with previous studies [12,13]. On the other hand, illustrative images displayed in Figure 5C,D correspond to macrophages cultured on human-derived DAMs obtained by the enzymatic method (hDAM1) and organic solvent treatment (hDAM2). In the presence of the fungus, macrophages on hDAMs exhibit long filopodia surrounding *C. albicans* yeast or hypha, as can be observed in Figure 5C,D, respectively. Conversely, macrophages cultured on pDAM1 and pDAM2, and in the presence of *C. albicans*, exhibit shorter actin protrusions (Figure 5E,F, respectively). It is important to note that, on porcine-derived DAMs, macrophages present a particular reorganization of their actin cytoskeleton, leading to the formation of a remarkable actin ring surrounding *C. albicans* hyphae. The formation of actin-rich structures has been reported during infection with other rod/filament-shaped microbes [61,62]. This actin cuff formation by epithelial cell lines in response to *C. albicans* has also been described by other authors [63,64,65]. Interestingly, macrophages, after contact with the fungus, rarely form actin cuffs surrounding the hypha. Only in Dectin-1 transfected macrophages, or when *C. albicans* hyphae are serum-opsonized, these F-actin structures are detected [66].

In summary, the tissue source, human or porcine, to obtain the DAMs determines the macrophage actin reorganization in response to *Candida*. There is evidence to suggest that this reorganization will depend on the biochemical composition of each DAM and more directly on the proteins that have been conserved. Furthermore, macrophages cultured on porcine-derived DAMs exhibited a newfangled and remarkable presence of actin cuffs during *Candida* interaction.

Macrophages are motile cells that dynamically alter their actin cytoskeleton to drive their migration towards the engulfment of pathogens, filopodia being the actin-based structures directly related to migration and interaction with pathogens. In this sense, different studies have demonstrated the influence of the integrated actin cytoskeleton on nuclear integrity and chromatin organization. Chromatin condensation has a structural role in supporting nuclear movement and/or changes in the morphology of the nucleus and is required for efficient cell migration [67]. Specifically, the actin cytoskeleton differentially alters the dynamics of lamin A, HP1α and H2B core histone proteins to remodel the chromatin condensation state in living cells [68]. Moreover, Xie et al. recently highlighted that in the eukaryotic cell nucleus, cytoskeletal proteins are emerging as essential players in nuclear function because actin regulates chromatin as part of ATP-dependent chromatin remodeling complexes [69]. We have analyzed the chromatin condensation of macrophages, cultured on human or porcine-derived DAMs and in the presence of *C. albicans*, to stablish a possible relationship with the actin cytoskeletal reorganization previously described (Figure 3 and Figure 5).

**Figure 6:**
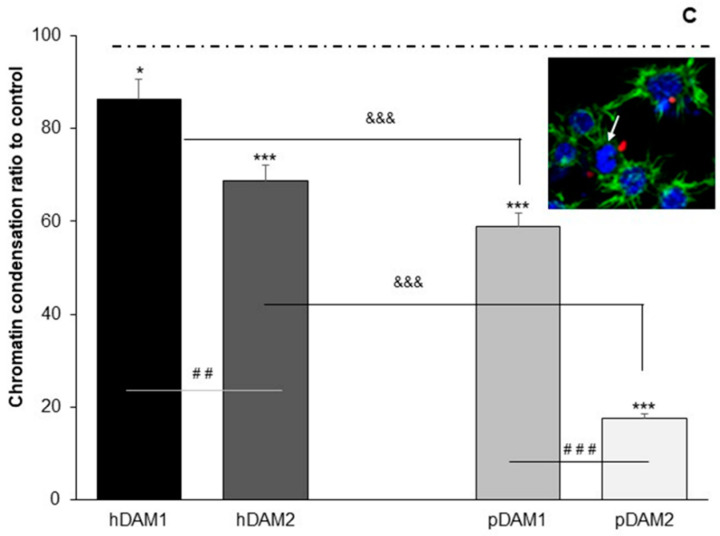
Chromatin condensation of RAW-264.7 macrophages cultured on the control surface (horizontal line C) and human (h) and porcine (p) DAMs obtained by the enzymatic method (hDAM1and pDAM1) and organic solvents treatment (hDAM2 and pDAM2) in presence of *C. albicans* (MOI 5). Inset: representative image of the chromatin condensation of RAW-264.7 macrophages on hDAM1 after *C. albicans* interaction. Statistical significance: *: *p* < 0.05; ***: *p* < 0.005 (comparison with respect to the control condition). ##: *p* < 0.01; ###: *p* < 0.005 (hDAM1 vs. hDAM2, pDAM1 vs. pDAM2). &&&: *p* < 0.005 (hDAM1 vs. pDAM1, hDAM2 vs. pDAM2).

Figure 6 shows the ratio of macrophages with condensed chromatin on the four DAMs after *Candida albicans* interaction with respect to the number of control macrophages (cultured on coverslips), with condensed chromatin considered as 100%. A representative image of a macrophage with condensed chromatin is also displayed (inset). The results indicate that when macrophages were cultured on all these DAMs, a lower number of cells with condensed chromatin was observed compared to control macrophages; this effect being more pronounced on pDAM2. In addition, it is important to highlight that there are statistically significant differences when comparing matrices from the same origin of the tissue (human or porcine) but after a different method of decellularization; that is, hDAM1 vs. hDAM2 and pDAM1 vs. pDAM2. In both cases, a lower number of cells with condensed chromatin was observed on the DAMs obtained after treatment with organic solvents (2) compared to enzymatic methods (1). It is also important to remark that there are statistically significant differences after comparing the human or porcine origin of the matrix regardless of the method of decellularization. Furthermore, the lowest number of macrophages with condensed chromatin was observed in porcine DAMs.

These results evidence a close relationship among the macrophage filopodia length (Figure 4) and the chromatin condensation (Figure 6), depending on the type of DAM to which the macrophages have been exposed. Thus, macrophages cultured on human DAMs exhibit a higher chromatin condensation and long filopodia, suggesting a higher ability to adhere to these human substrates. On the other hand, macrophages on porcine DAMs present short filopodia (Figure 4) and a lower chromatin condensation (compared to human DAMs), being more pronounced in pDAM2. In this sense, we want to highlight that these two events could be related to the observed effect of these DAMs on macrophage/*Candida albicans* interaction, thus rendering a different reorganization of the phagocytic actin cytoskeleton surrounding the fungus (Figure 5).

### 3.4. TNFα and IL-6 Production by RAW-264.7 Macrophages Cultured on Human or Porcine DAMs after Candida albicans Interaction

Macrophages exhibit functional plasticity characterized by different cell surface markers and cytokines that allows them to polarize towards pro-inflammatory (M1) and anti-inflammatory (M2) macrophages. M1 macrophages are characterized by tumor necrosis factor-alpha (TNF-α) and IL-6 release. TNFα is considered a “master regulator” of proinflammatory cytokine production [70]. IL-6 is a pleiotropic inflammatory cytokine that has emerged as a potent regulator of immune responses and inflammation [71].

**Figure 7:**
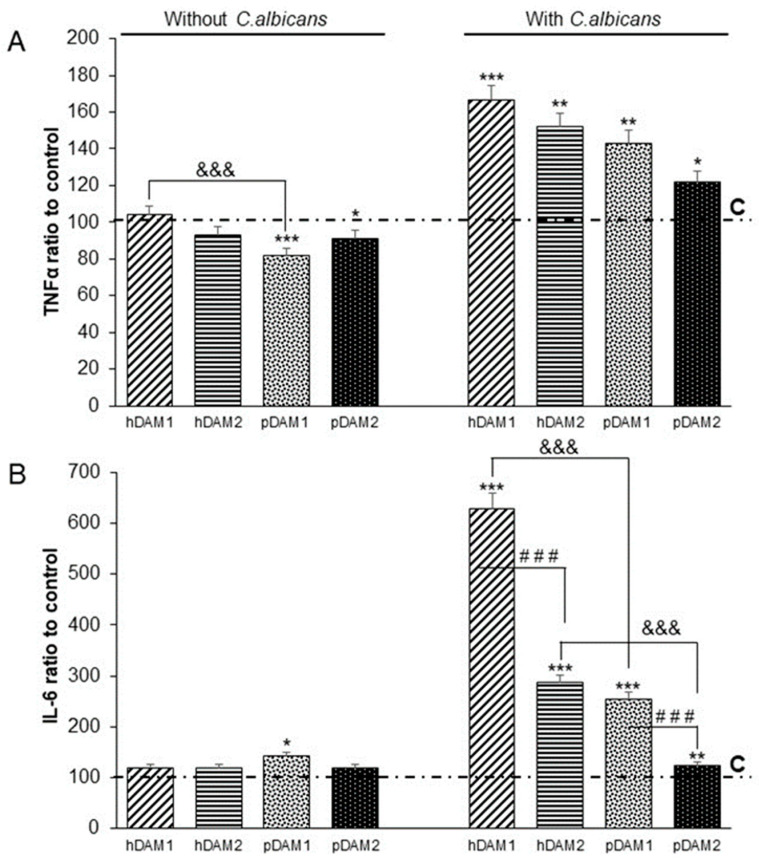
TNFα (**A**) and IL-6 (**B**) production by RAW-264.7 macrophages cultured on control surface (horizontal line C) and human (h) and porcine (p) DAMs obtained by the enzymatic method (hDAM1and pDAM1) and organic solvents treatment (hDAM2 and pDAM2) in the presence or absence of *C. albicans* (MOI5). Statistical significance, *: *p* < 0.05; **: *p* < 0.01; ***: *p* < 0.005 (comparison with respect to the control condition). ###: *p* < 0.005 (hDAM1 vs. hDAM2, pDAM1 vs. pDAM2). &&&: *p* < 0.005 (hDAM1 vs. pDAM1, hDAM2 vs. pDAM2).

Figure 7 displays the TNFα (A) and IL-6 (B) production by macrophages cultured on human or porcine-derived DAMs obtained by the enzymatic method (hDAM1 and pDAM1) and organic solvents treatment (hDAM2 and pDAM2) in the presence or absence of *C. albicans.* Control macrophages are macrophages cultured on glass coverslips in the absence or in the presence of *C. albicans* and the values of these controls have been taken as reference and are considered in each case as 100%. The cytokine levels ratio produced by macrophages after culture on the different DAMs is shown with respect to the level of cytokines released by control macrophages.

Concerning TNFα production (Figure 7A), when macrophages were cultured on all the DAMs and in absence of the fungus, it should be noted that statistically lowest levels of TNFα were obtained in macrophages cultured on matrices of porcine origin (pDAM1 and pDAM2). As expected, when macrophages were cultured on all the matrices and in the presence of the fungus *C. albicans*, a significant increase in all the TNFα values were detected. These results demonstrate the macrophage activation induced by the fungus. On the other hand, the authors want to highlight that the cytokine levels produced by macrophages depend on the type of matrix on which they have been previously cultured (hDAM1 > hDAM2 > pDAM1 > pDAM2).

Regarding cytokine IL-6 (Figure 7B), macrophages cultured on the different DAMs, and in absence of *C. albicans*, produce similar levels of this cytokine as macrophages cultured on the control surfaces. Conversely, when macrophages were cultured on the different matrices and then exposed to *C. albicans*, a significant increase in the IL-6 levels was observed, with the macrophages that adhered to hDAM1 being those with the highest IL-6 production (six-fold to control). Moreover, significant differences on the Il-6 secreted by macrophages cultured on the different DAMs depend on the origin of the tissue (human or porcine) as well as the decellularization method applied. In this sense, we have detected that macrophages cultured on hDAMs produced higher IL-6 levels than pDAMs (hDAM1 and hDAM2 vs. pDAM1 and pDAM2, respectively). Furthermore, regarding the decellularized method applied to DAMs, we also observed that macrophages cultured on DAMs obtained by the enzymatic treatment showed a higher production of this pro-inflammatory cytokine compared to macrophages that adhered to DAMs generated by organic solvents (hDAM1 vs. hDAM2 and pDAM1 vs. pDAM2).

Evidence has also emerged that the ECM conveys specific signals to cells, thereby modulating essential immune functions such as cell migration, activation, proliferation and differentiation [72]. Therefore, the biochemical composition of the ECM, including specific proteins and lipids, will play a key role in all these macrophage functions as well as in their metabolism. The different lipid content of these four DAMs has been described in Section 3.1, being dependent on the tissue source and decellularization method employed. In this context, the lipid metabolism of macrophages plays an important role in their activation and appropriate response to any stimulus from the extracellular microenvironment [18]. Thus, it has been evidenced that the fatty acid esterification and storage in lipid droplets prevent pro-inflammatory macrophage activation [19,20]. On the other hand, fatty acid oxidation is linked to the M2 macrophage state because it has been evidenced that interleukin-4 (IL-4) stimulates this metabolic pathway during the reparative response of macrophages, producing acetyl-CoA to promote histone acetylation and expression of IL-4-inducible genes [21]. In this work we demonstrate that porcine-derived DAMs, especially pDAM2, produce a significant decrease in TNF-α and IL6 levels in the presence of the fungus compared to human-derived DAMs. This effect could be related to the higher triglyceride content of porcine-derived DAMs [19], which will influence the intracellular concentration of lipids in macrophages and the possible use of fatty acids as an energy source, inducing a more reparative response on porcine-derived DAMs. Considering both the TNFα and IL-6 levels produced by macrophages cultured on the different DAMs, and in absence of the fungus, we can conclude that the DAMs studied in this work do not exert a pro-inflammatory effect on macrophages. These results agree with our previous studies recently published, in which specific markers of macrophage phenotypes M1 and M2 were evaluated [34]. In these previous studies, we observed that porcine matrices induced the M2 reparative phenotype in macrophages as detected by CD163 and CD206 expression and low intracellular ROS content. These data could be also related to the lower proinflammatory cytokine secretion (TNFα and IL-6) shown in the present study on porcine DAMs. In this sense, we want to highlight that the increased phagocytosis observed in macrophages cultured on pDAM2 could be explained not only by the polarization towards the reparative phenotype of macrophages [73,74] but also by the presence of differentially preserved proteins detected in this pDAM2. Further studies should be addressed in order to explain the relationship of these actin cuffs surrounding *C. albicans* after macrophage exposure to pDAM2, and the increased phagocytosis previously described.

On the other hand, recent data link an active role of the ECM in the immune response to infection, encompassing antimicrobial activities, microbial recognition, macrophage activation and transcriptional and post-transcriptional regulation of inflammatory networks. Thus, the ECM and the immune system are intertwined: signals from the ECM help to coordinate immune responses and, in turn, immune cells promote ECM repair and regeneration through the release of cytokines such as TNFα, interferon-γ and TGF-β, which regulate the expression of many ECM molecules [75].

Due to the importance of the ECM–cell communication, the employment of in vitro models mimicking a fungal infection scenario is essential to evaluate the ECM effects on the macrophage response to the fungus. In the present work, the study of the *C. albicans*/macrophage interface has allowed us to detect the differences in the activation of macrophages cultured on human- and porcine-derived DAMs, focusing on aspects related to the macrophage actin cytoskeleton and its reorganization against the fungus. Moreover, the cell morphological changes observed on these four DAMs are key to understand the functionality of the macrophages as defense cells against fungal pathogens, depending on their extracellular microenvironment. Thus, far from being static studies, our in vitro model with macrophages, *Candida albicans* and the extracellular matrix offers a dynamic picture with multiple possibilities of investigation.

## Figures and Tables

**Table 1:** Main DAM proteins involved in cell adhesion.

PROTEINS	DAMs			
	hDAM1	hDAM2	pDAM1	pDAM2
**Collagens**				
Collagen α-1(I) chain	✓	✓	✓	✓
Collagen α-1(III) chain	✓	✓		
Collagen α-1(IV) chain	✓	✓		
Collagen α-1(V) chain	✓	✓		
Collagen α-1(VI) chain	✓	✓	✓	
Collagen α-1(XII) chain	✓	✓	✓	✓
Collagen α-1(XIV) chain	✓	✓	✓	✓
Collagen α-1(XV) chain	✓	✓	✓	✓
Collagen α-1(XVIII) chain	✓	✓		
Collagen α-2 (I) chain	✓	✓		
Collagen α-2 (IV) chain	✓	✓		✓
Collagen α-2 (V) chain	✓	✓		✓
Collagen α-2(VI) chain	✓	✓	✓	✓
Collagen α-3 (V) chain	✓	✓		
Collagen α-3 (VI) chain	✓	✓	✓	✓
**Laminins**				
Laminin α-2	✓	✓		
Laminin α-3	✓			
Laminin α-4	✓	✓		
Laminin α-5	✓	✓		
Laminin β-1	✓	✓	✓	✓
Laminin β-2	✓	✓	✓	✓
Laminin γ-1	✓	✓		✓
**EMILIN**				
Emilin-1	✓	✓		
**Fibulins**				
Fibulin-1		✓		
Fibulin-2	✓	✓		
Fibulin-5	✓	✓		✓
**von Willebrand factor**	✓	✓		

## Data Availability

Data is contained within the article.

## References

[1] Hebecker B., Naglik J.R., Hube B., Jacobsen I.D. (2014). Pathogenicity mechanisms and host response during oral *Candida albicans* infections. Expert Rev. Anti-Infect. Ther..

[2] Pfaller M.A., Diekema D.J. (2007). Epidemiology of invasive candidiasis: A persistent public health problem. Clin. Microbiol. Rev..

[3] Kullberg B.J., Arendrup M.C. (2015). Invasive Candidiasis. N. Engl. J. Med..

[4] Sudbery P., Gow N., Berman J. (2004). The distinct morphogenic states of *Candida albicans*. Trends Microbiol..

[5] Cassone A. (2015). Vulvovaginal Candida albicans infections: Pathogenesis, immunity and vaccine prospects. BJOG..

[6] Chaffin W.L. (2008). *Candida albicans* cell wall proteins. Microbiol. Mol. Biol. Rev..

[7] Moyes D.L., Richardson J.P., Naglik J.R. (2015). *Candida albicans*-epithelial interactions and pathogenicity mechanisms: Scratching the surface. Virulence.

[8] Murray P.J., Allen J.E., Biswas S.K., Fisher E.A., Gilroy D.W., Goerdt S., Gordon S., Hamilton J.A., Ivashkiv L.B., Lawrence T. (2014). Macrophage activation and polarization: Nomenclature and experimental guidelines. Immunity.

[9] Mantovani A., Sica A., Sozzani S., Allavena P., Vecchi A., Locati M. (2004). The chemokine system in diverse forms of macrophage activation and polarization. Trends Immunol..

[10] Luig M., Kluger M.A., Goerke B., Meyer M., Nosko A., Yan I., Scheller J., Mittrücker H.W., Rose-John S., Stahl R.A. (2015). Inflammation-Induced IL-6 Functions as a Natural Brake on Macrophages and Limits GN. J. Am. Soc. Nephrol..

[11] Gubernatorova E.O., Gorshkova E.A., Namakanova O.A., Zvartsev R.V., Hidalgo J., Drutskaya M.S., Tumanov A.V., Nedospasov S.A. (2018). Non-redundant Functions of IL-6 Produced by Macrophages and Dendritic Cells in Allergic Airway Inflammation. Front. Immunol..

[12] Rougerie P., Miskolci V., Cox D. (2013). Generation of membrane structures during phagocytosis and chemotaxis of macrophages: Role and regulation of the actin cytoskeleton. Immunol. Rev..

[13] Barger S.R., Reilly N.S., Shutova M.S., Li Q., Maiuri P., Heddleston J.M., Mooseker M.S., Flavell R.A., Svitkina T., Oakes P.W. (2019). Membrane-cytoskeletal crosstalk mediated by myosin-I regulates adhesion turnover during phagocytosis. Nat. Commun..

[14] Lorenz M.C., Bender J.A., Fink G.R. (2004). Transcriptional response of *Candida albicans* upon internalization by macrophages. Eukaryot. Cell.

[15] Fernández-Arenas E., Cabezón V., Bermejo C., Arroyo J., Nombela C., Diez-Orejas R., Gil C. (2007). Integrated proteomics and genomics strategies bring new insight into *Candida albicans* response upon macrophage interaction. Mol. Cell. Proteomics.

[16] Erwig L.P., Gow N.A.R. (2016). Interactions of fungal pathogens with phagocytes. Nat. Rev. Microbiol..

[17] Lewis L.E., Bain J.M., Lowes C., Gillespie C., Rudkin F.M., Gow N.A., Erwig L.P. (2012). Stage specific assessment of *Candida albicans* phagocytosis by macrophages identifies cell wall composition and morphogenesis as key determinants. PLoS Pathog..

[18] Jung J., Zeng H., Horng T. (2019). Metabolism as a guiding force for immunity. Nat. Cell Biol..

[19] Yan J., Horng T. (2020). Lipid metabolism in regulation of macrophage functions. Trends Cell Biol..

[20] Huang S.C., Everts B., Ivanova Y., O’sullivan D., Nascimento M., Smith A.M., Beatty W., Love-Gregory L., Lam W.Y., O’neill C.M. (2014). Cell-intrinsic lysosomal lipolysis is essential for alternative activation of macrophages. Nat. Immunol..

[21] Pandey A.K., Sassetti C.M. (2008). Mycobacterial persistence requires the utilization of host cholesterol. Proc. Natl. Acad. Sci. USA.

[22] Frantz C., Stewart K.M., Weaver V.M. (2010). The extracellular matrix at a glance. J. Cell Sci..

[23] Badylak S.F. (2014). Decellularized allogeneic and xenogeneic tissue as a bioscaffold for regenerative medicine: Factors that influence the host response. Ann. Biomed. Eng..

[24] Sasikumar S., Chameettachal S., Cromer B., Pati F., Kingshott P. (2019). Decellularized extracellular matrix hydrogels—Cell behavior as a function of matrix stiffness. Curr. Opin. Biomed. Eng..

[25] Abaricia J.O., Shah A.H., Chaubal M., Hotchkiss K.M., Olivares-Navarrete R. (2020). Wnt signaling modulates macrophage polarization and is regulated by biomaterial surface properties. Biomaterials.

[26] Yu C., Kornmuller A., Brown C., Hoare T., Flynn L. (2017). Decellularized adipose tissue microcarriers as a dynamic culture platform for human adipose-derived stem/stromal cell expansion. Biomaterials.

[27] Ibsirlioglu T., Elçin A.E., Elçin Y.M. (2020). Decellularized biological scaffold and stem cells from autologous human adipose tissue for cartilage tissue engineering. Methods.

[28] Keane T.J., Swinehart I.T., Badylak S.F. (2015). Methods of tissue decellularization used for preparation of biologic scaffolds and in vivo relevance. Methods.

[29] Dominic M., Handleton R., Shazly T., Matthews M. (2018). A novel supercritical CO_2_-based decellularization method for maintaining scaffold hydration and mechanical properties. J. Supercrit. Fluids.

[30] Hussey G.S., Dziki J.L., Badylak S.F. (2018). Extracellular matrix-based materials for regenerative medicine. Nat. Rev. Mater..

[31] Dembo M., Torney D., Saxman K., Hammer D. (1988). The kinetics of membrane-to-surface adhesión and detachment. Proc. R. Soc..

[32] Khalili A.A., Ahmad M.R. (2015). A Review of Cell Adhesion Studies for Biomedical and Biological Applications. Int. J. Mol. Sci..

[33] Sagvolden G., Giaever I., Pettersen E.O., Feder J. (1999). Cell adhesion force microscopy. Proc. Natl. Acad. Sci. USA.

[34] Cicuéndez M., Casarrubios L., Feito M.J., Madarieta I., Garcia-Urkia N., Murua O., Olalde B., Briz N., Diez-Orejas R., Portolés M.T. (2021). Effects of Human and Porcine Adipose Extracellular Matrices Decellularized by Enzymatic or Chemical Methods on Macrophage Polarization and Immunocompetence. Int. J. Mol. Sci..

[35] Gillum A.M., Tsay E.Y., Kirsch D.R. (1984). Isolation of the *Candida albicans* gene for orotidine-5’-phosphate decarboxylase by complementation of *S. cerevisiae* ura3 and *E. coli* pyrF mutations. Mol. Gen. Genet..

[36] Shaner N.C., Campbell R.E., Steinbach P.A., Giepmans B.N., Palmer A.E., Tsien R.Y. (2004). Improved monomeric red, orange and yellow fluorescent proteins derived from *Discosoma* sp. red fluorescent protein. Nat. Biotechnol..

[37] Prieto D., Román E., Correia I., Pla J. (2014). The HOG pathway is critical for the colonization of the mouse gastrointestinal tract by *Candida albicans*. PLoS ONE.

[38] Yaoa Q., Zheng Y.-W., Lan Q.-H., Kou L., Xu H.L., Zhao Y.-Z. (2019). Recent development and biomedical applications of decellularized extracellular matrix biomaterials. Mater. Sci. Eng. C.

[39] Naba A., Clauser K.R., Ding H., Whittaker C.A., Carr S.A., Hynes R.O. (2016). The extracellular matrix: Tools and insights for the “omics” era. Matrix Biol..

[40] Mariani E., Lisignoli G., Borzì R.M., Pulsatelli L. (2019). Biomaterials: Foreign Bodies or Tuners for the Immune Response?. Int. J. Mol. Sci..

[41] McWhorter F.Y., Davis C.T., Liu W.F. (2015). Physical and mechanical regulation of macrophage phenotype and function. Cell Mol. Life Sci..

[42] Mattila P., Lappalainen P. (2008). Filopodia: Molecular architecture and cellular functions. Nat. Rev. Mol. Cell Biol..

[43] Niedergang F., Chavrier P. (2004). Signaling and membrane dynamics during phagocytosis: Many roads lead to the phagos(R)ome. Curr. Opin. Cell Biol..

[44] Kress H., Stelzer E.H., Holzer D., Buss F., Griffiths G., Rohrbach A. (2007). Filopodia act as phagocytic tentacles and pull with discrete steps and a load-dependent velocity. Proc. Natl. Acad. Sci. USA.

[45] Vonna L., Wiedemann A., Aepfelbacher M., Sackmann E. (2007). Micromechanics of filopodia mediated capture of pathogens by macrophages. Eur. Biophys. J..

[46] Koyama Y., Norose-Toyoda K., Hirano S., Kobayashi M., Ebihara T., Someki I., Fijisaki H., Irie S. (2000). Type I collagen is a non-adhesive extracellular matrix for macrophages. Arch. Histol. Cytol..

[47] Gowen B.B., Borg T.K., Ghaffar A., Mayer E.P. (2000). Selective adhesion of macrophages to denatured forms of type I collagen is mediated by scavenger receptors. Matrix Biol..

[48] Mazur A., Holthoff E., Vadali S., Kelly T., Post S.R. (2016). Cleavage of Type I Collagen by Fibroblast Activation Protein-α Enhances Class A Scavenger Receptor Mediated Macrophage Adhesion. PLoS ONE.

[49] Pöschl E., Schlötzer-Schrehardt U., Brachvogel B., Saito K., Ninomiya Y., Mayer U. (2004). Collagen IV is essential for basement membrane stability but dispensable for initiation of its assembly during early development. Development.

[50] Bahr J.C., Weiss S.J. (2018). Macrophage-Dependent Tracking and Remodeling of the Basement Membrane-Interstitial Matrix Interface. BioRxiv.

[51] Etich J., Koch M., Wagener R., Zaucke F., Fabri M., Brachvogel B. (2019). Gene Expression Profiling of the Extracellular Matrix Signature in Macrophages of Different Activation Status: Relevance for Skin Wound Healing. Int. J. Mol. Sci..

[52] Simon T., Bromberg J.S. (2017). Regulation of the immune system by laminins. Trends Immunol..

[53] Shaw L.M., Messier J.M., Mercurio A.M. (1990). The activation dependent adhesion of macrophages to laminin involves cytoskeletal anchoring and phosphorylation of the α6β1 integrin. J. Cell Biol..

[54] Iorio V., Troughton L.D., Hamill K.J. (2015). Laminins: Roles and Utility in Wound Repair. Adv. Wound Care.

[55] Ishihara J., Ishihara A., Fukunaga K., Sasaki K., White M.J.V., Briquez P.S., Hubbell J.A. (2018). Laminin heparin-binding peptides bind to several growth factors and enhance diabetic wound healing. Nat. Commun..

[56] Schiavinato A., Keene D.R., Wohl A.P., Corallo D., Colombatti A., Wagener R., Paulsson M., Bonaldo P., Sengle G. (2016). Targeting of EMILIN-1 and EMILIN-2 to Fibrillin Microfibrils Facilitates their Incorporation into the Extracellular Matrix. J. Investig. Dermatol..

[57] Yanagisawa H., Davis E.C., Starcher B.C., Ouchi T., Yanagisawa M., Richardson J.A., Olson E.N. (2002). Fibulin-5 is an elastin-binding protein essential for elastic fibre development in vivo. Nature.

[58] Wohner N., Muczynski V., Mohamadi A., Legendre P., Proulle V., Aymé G., Christophe O.D., Lenting P.J., Denis C.V., Casari C. (2018). Macrophage scavenger receptor SR-AI contributes to the clearance of von Willebrand factor. Haematologica.

[59] Adair-Kirk T.L., Senior R.M. (2008). Fragments of extracellular matrix as mediators of inflammation. Int. J. Biochem. Cell Biol..

[60] Boyd D.F., Thomas P.G. (2017). Towards integrating extracellular matrix and immunological pathways. Cytokine.

[61] Gerisch G., Ecke M., Schroth-Diez B., Gerwig S., Engel U., Maddera L., Clarke M. (2009). Self-organizing actin waves as planar phagocytic cup structures. Cell Adhes. Migr..

[62] Prashar A., Bhatia S., Gigliozzi D., Martin T., Duncan C., Guyard C., Terebiznik M.R. (2013). Filamentous morphology of bacteria delays the timing of phagosome morphogenesis in macrophages. J. Cell Biol..

[63] Ashwini N.A., Sachin V.S., Yogesh S.S., Jomon J., Milind S.P., Rajendra L.D. (2009). Association of small Rho GTPases and actin ring formation in epithelial cells during the invasion by *Candida albicans*. FEMS Immunol. Med. Microbiol..

[64] Tsarfaty I., Sandovsky-Losica H., Mittelman L., Berdicevsky I., Segal E. (2000). Cellular actin is affected by interaction with *Candida albicans*. FEMS Microbiol. Lett..

[65] Sandovsky-Losica H., Segal E. (2006). Infection of Hep2 epithelial cells with *Candida albicans*: Adherence and postadherence events. FEMS Immunol. Med. Microbiol..

[66] Maxson M.E., Naj X., O’Meara T.R., Plumb J.D., Cowen L.E., Grinstein S. (2018). Integrin-based diffusion barrier separates membrane domains enabling the formation of microbiostatic frustrated phagosomes. ELife.

[67] Gerlitz G., Bustin M. (2010). Efficient cell migration requires global chromatin condensation. J. Cell Sci..

[68] Chua T.K., Ramdas N.M., Shivashankar G.V. (2015). Actin cytoskeleton differentially alters the dynamics of lamin A, HP1α and H2B core histone proteins to remodel chromatin condensation state in living cells. Integr. Biol..

[69] Xie S.X., Mahmood R., Gjorgjieva T., Percipalle P. (2020). Emerging roles of cytoskeletal proteins in regulating gene expression and genoma organization during differentiation. Nucleus.

[70] Parameswaran N., Patial S. (2010). Tumor necrosis factor-α signaling in macrophages. Crit. Rev. Eukaryot. Gene Expr..

[71] Choy E., Rose-John S. (2017). Interleukin-6 as a multifunctional regulator: Inflammation, immune response, and fibrosis. J. Scleroderma Relat. Disord..

[72] Luu T.U., Liu W.F. (2018). Regulation of Macrophages by Extracellular Matrix Composition and Adhesion Geometry. Regen. Eng. Transl. Med..

[73] Reales-Calderón J.A., Aguilera-Montilla N., Corbí A.L., Molero G., Gil C. (2014). Proteomic characterization of human proinflammatory M1 and anti-inflammatory M2 macrophages and their response to *Candida albicans*. Proteomics.

[74] Jaggi U., Yang M., Matundan H.H., Hirose S., Shah P.K., Sharifi B.G., Ghiasiet H. (2020). Increased phagocytosis in the presence of enhanced M2-like macrophage responses correlates with increased primary and latent HSV-1 infection. PLoS Pathog..

[75] Tomlin H., Piccinini A.M. (2018). A complex interplay between the extracellular matrix and the innate immune response to microbial pathogens. Immunology.

